# Negative correlation of ITCH E3 ubiquitin ligase and miRNA-106b dictates metastatic progression in pancreatic cancer

**DOI:** 10.18632/oncotarget.6395

**Published:** 2015-11-26

**Authors:** Zhu-lin Luo, Hui-jun Luo, Chen Fang, Long Cheng, Zhu Huang, Ruiwu Dai, Kun Li, Fu-zhou Tian, Tao Wang, Li-jun Tang

**Affiliations:** ^1^ Department of General Surgery, Chengdu Military General Hospital, Chengdu, Sichuan 610083, P. R. China; ^2^ Department of Biochemistry and Molecular Biology, Mayo Clinic Arizona, Scottsdale, AZ 85259, USA; ^3^ Chengdu Military Institute for Drug and Instrument Control, Chengdu, Sichuan 610020, P. R. China; ^4^ Medical Central Laboratory, Chengdu Military General Hospital, Chengdu, Sichuan 610083, P. R. China

**Keywords:** ITCH, miR-106b, pancreatic cancer, YAP, metastasis

## Abstract

Pancreatic cancer is one of the major malignancies and cause for mortality across the world, with recurrence and metastatic progression remaining the single largest cause of pancreatic cancer mortality. Hence it is imperative to develop novel biomarkers of pancreatic cancer prognosis. The E3 ubiquitin ligase ITCH has been previously reported to inhibit the tumor suppressive Hippo signaling by suppressing LATS1/2 in breast cancer and chronic lymphocytic leukemia. However, the role of ITCH in pancreatic cancer progression has not been described. Here we report that *ITCH* transcript and protein expression mimic metastatic trait in pancreatic cancer patients and cell lines. Loss-of-function studies of *ITCH* showed that the gene product is responsible for inducing metastasis *in vivo*. We furthermore show that *hsa-miR-106b*, which itself is down regulated in metastatic pancreatic cancer, directly interacts and inhibit *ITCH* expression. *ITCH* and *hsa-miR-106b* are thus potential biomarkers for pancreatic cancer prognosis.

## INTRODUCTION

Pancreatic cancer is one of the major malignancies and cause for mortality across the world with an estimated 227000 deaths per year worldwide [1]. Tumor metastasis is responsible for over 90% of cancer mortality [2, 3]. In fact, pancreatic ductal adenocarcinoma (PDAC) is the most common histological type that presents with a highly invasive and metastatic phenotype, and is often responsible for treatment failure and poor clinical prognosis. Insightful knowledge about regulatory mechanisms underlying pancreatic cancer metastasis is required to define novel diagnostic and/or prognostic markers.

Impairment of the Hippo signaling pathway has been indicated in tumor development and metastasis of different cancer types [4, 5]. The Hippo pathway is composed of a kinase cascade, inclusive of MST1/2 serine/threonine kinase (ortholog of Hpo in *Drosophila melanogaster*), WW45 scaffold protein (Sav), MOB (Mats) and large tumor suppressor (LATS) 1 and 2 kinases (Wts) [4]. The *Drosophila* LATS is now recognized as a major component of a tumor suppressor pathway known as the Hippo-LATS pathway [4, 6, 7]. In this pathway, LATS transmits signals from upstream tumor suppressor proteins (FAT, Merlin, Expanded, Salvador, RASSF, Hippo, and MATS) to inhibit tumor growth by phosphorylating and suppressing the oncoprotein and transcriptional coactivator Yorkie [4, 7]. The mammalian homologue of Yorkie is YAP (Yes-associated protein) and TAZ (transcriptional coactivator with PDZ-binding motif) [7]. In fact, a similar tumor suppressor role for LATS1/2 has now been established in mammalian cells [4]. LATS1/2 mediated phosphorylation of YAP or TAZ prevents YAP and TAZ translocation to the nucleus by causing β-TRCP-dependent proteasomal degradation [8]. This in turn inhibits transcription of downstream target genes that cumulatively would have promoted a pro-cancerous phenotype [9].

However, two separate studies showed that the Itch E3 ubiquitin ligase is a bona fide binding partner and negative regulator of LATS1 [10, 11] and was accompanied by YAP accumulation and translocation into the nucleus thus indeed phenocopying YAP activation [10, 11]. ITCH belongs to the NEDD4-like family of E3 ubiquitin ligases and contains 4 WW domains (that associate with PPxY containing targets, conferring substrate specificity), and a HECT-type ligase domain that renders the catalytic E3 activity [12]. Multiple substrates of ITCH has been identified, inclusive of LATS1 [11], p63 [13], p73 [14], ErbB4 [15], and c-Jun [16, 17]. In fact, a positive correlation between ITCH and tumor progression has been suggested in breast cancer [18], and chronic lymphocytic leukemia [19]. However, levels of ITCH expression, its correlation to LATS1 levels and function, and its regulation has not yet been established in the context of pancreatic cancer.

In the present study, we analyzed *LATS1* and *ITCH* expression in pancreatic tumor tissue specimens compared to normal pancreatic tissue specimens and correlated the expression levels to overall survival and percent disease progression. Functional consequence of *ITCH* expression on pancreatic cancer metastasis was confirmed using *in vivo* animal models of experimental metastasis. We next determined that *has-miR-106b* targets *ITCH* transcript and that differential expression of *has-miR-106b* determines expression level of *ITCH* in non-metastatic and metastatic pancreatic cancer cell lines and patients. Cumulatively, our data indicates that the inverse correlation between ITCH and *has-miR-106b* is associated with metastasis in human pancreatic cancer.

## RESULTS

### ITCH expression is upregulated in pancreatic cancer tissues and correlates with poorer survival

**Figure 1:**
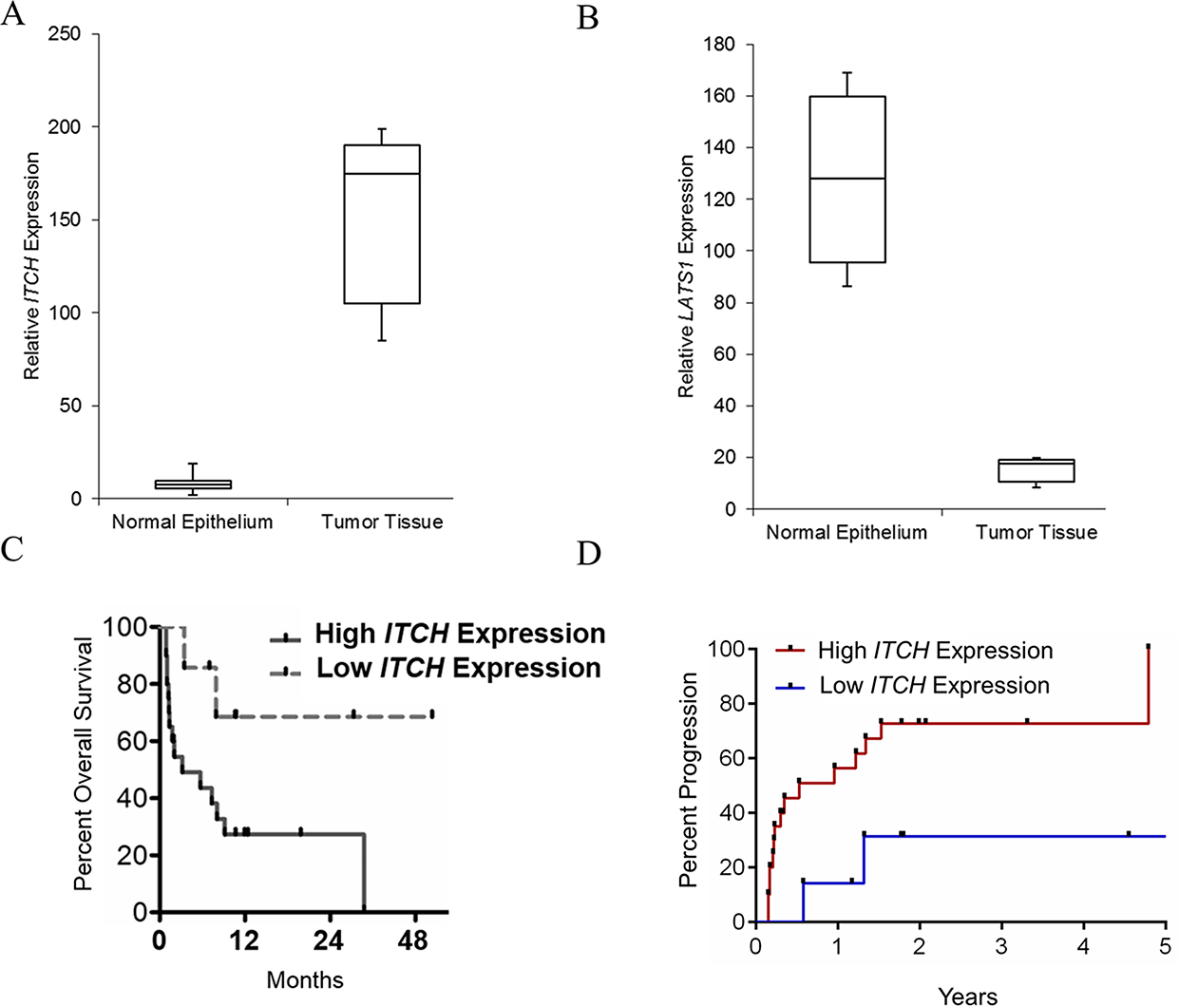
ITCH levels positively correlate with metastatic pancreatic cancer (**A**, **B**) Steady state expression of *LATSI* (A) and *ITCH* (B) mRNA was determined. Data was normalized to *TBP* expression. *P < 0.05. (B) Kaplan–Meier overall survival curves based on differential *ITCH* mRNA expression level. The overall survival of the High-*ITCH* group (*n* = 15: *ITCH* expression ratio ≥ median ratio) was significantly higher than that of Low-*ITCH* group (*n* = 15; *ITCH* expression ratio ≤ median ratio; *P* = 0.0039, log-rank test). (**C**, **D**), (B) Kaplan–Meier overall survival curves (C) and percent progression (D) based on differential *ITCH* mRNA expression level.

**Figure 2:**
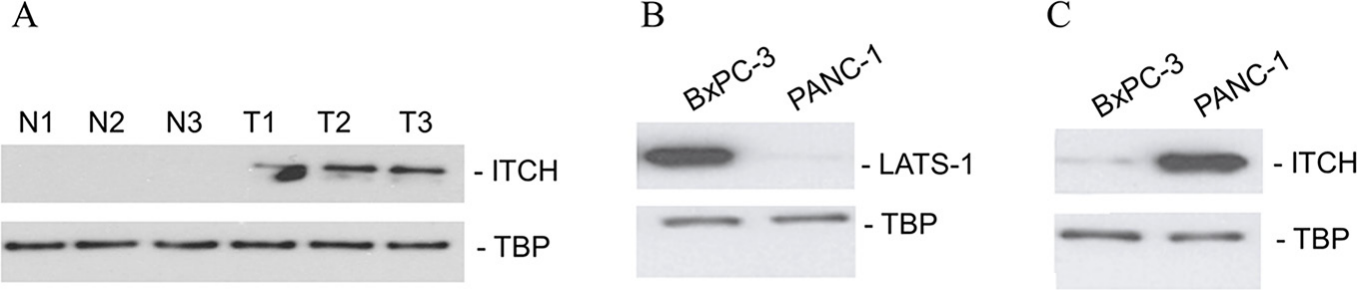
ITCH protein expression correlate with metastatic potential in patient samples and in cell lines (**A**) Immunoblot analysis of ITCH in three matched normal-tumor tissue specimens from pancreatic cancer patients. (**B**, **C**) Immunoblot analysis of ITCH and LATS-1 in the non-metastatic BxPC-3 and metastatic PANC-1 cell line. TBP served as a loading control in each case.

The level of *ITCH* and *LATS1* expression were determined in 30 paired pancreatic cancer samples and matched adjacent, histologically normal tissues by qRT-PCR, and normalized to *TBP* expression (internal control). Whereas, *ITCH* expression was significantly upregulated in cancerous tissues (mean ratio of 143.14-fold, *P* < 0.01) compared with normal counterparts (Figure 1A), *LATS1* expression was significantly low (mean ration of 11.23, *P* < 0.005) (Figure 1B). *ITCH* and *LATS1* expression were not associated with gender (*P* = 0.634) and tumor site (*P* = 1.339). However, *ITCH* expression was significantly associated with tumor cell differentiation (*P* = 0.018) and distant metastasis (*P* = 0.001). Furthermore, the patient cohort with relatively higher *ITCH* expression had a significantly less overall survival (*P* = 0.036) (Figure 1C) and a significantly higher percent progression (*P* = 0.0039) compared to the cohort with relatively lower *ITCH* expression (Figure 1D), cumulatively reinforcing that *ITCH* expression is upregulated in metastatic pancreatic cancer and might be useful as a diagnostic and prognostic marker for pancreatic cancer. The difference in *ITCH* transcript expression between normal and tumorigenic pancreatic tissue was corroborated at the protein expression level (Figure 2A).

### ITCH's expression correlates with metastatic potential of pancreatic cancer cell lines

We next determined the steady state LATS1 and ITCH protein expression levels in the non-metastatic pancreatic cancer cell line, BxPC-3 [20] and the metastatic pancreatic cancer cell line, PANC-1 [20]. LATS1 expression was downregulated in the metastatic PANC-1, but not the non-metastatic BxPC-3 cell line (Figure 2B). ITCH expression showed a converse relationship to LATS-1 expression and was higher in the metastatic PANC-1 cell line (Figure 2C). The results indicated that ITCH protein expression mimicked the metastatic (or mesenchymal state) of the cells and also gave us a model system where the role of ITCH and mechanism of its regulation in pancreatic cancer could be further investigated.

### ITCH is essential for pancreatic cancer metastasis *in vivo*

**Figure 3:**
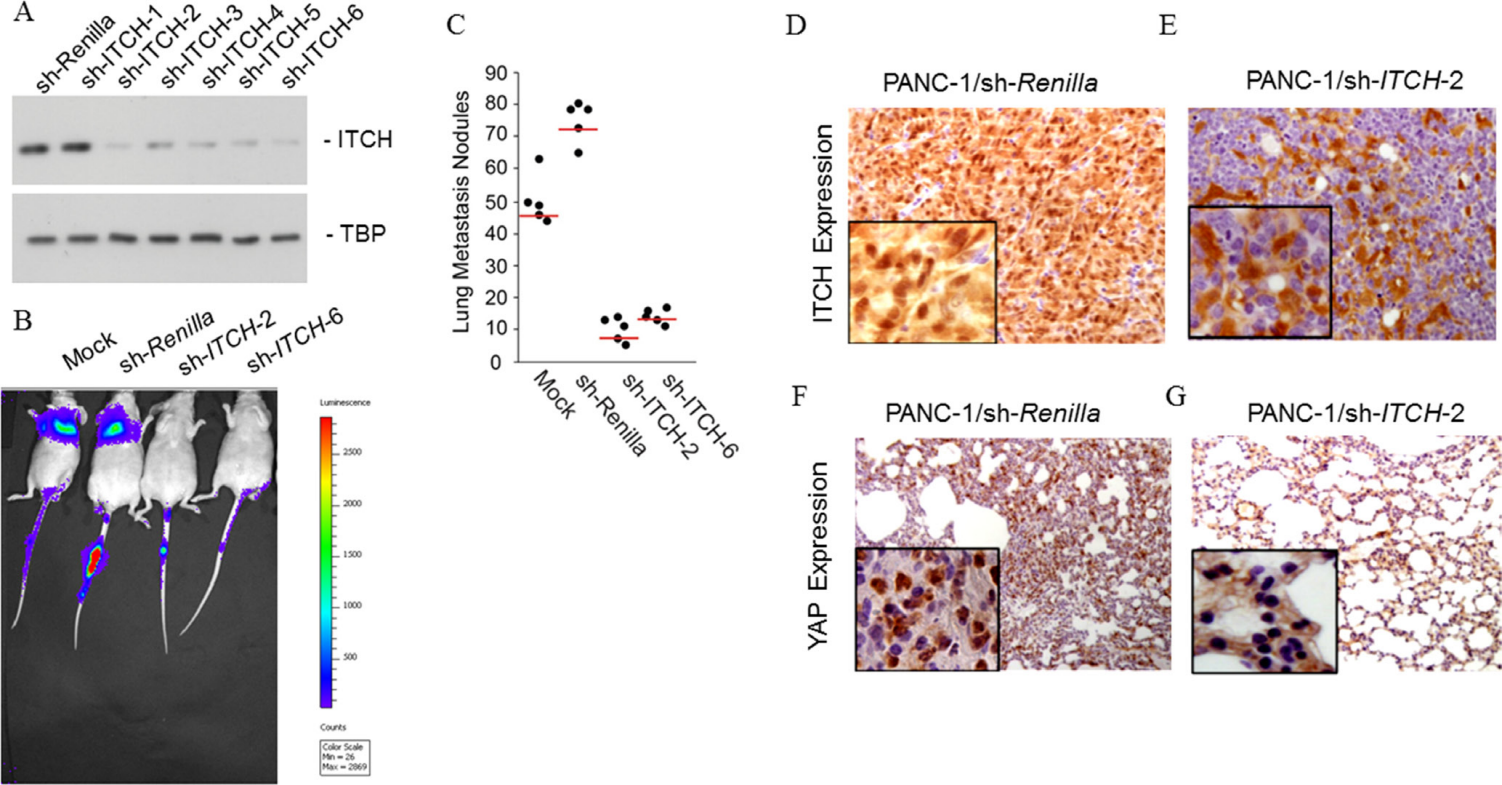
*ITCH* drive metastasis *in vivo* (**A**) Immunoblot analysis to confirm successful shRNA-mediated silencing of ITCH protein expression in PANC-1 cells. TBP served as a loading control. (**B**) Firefly luciferase expression PANC-1 cells stably expressing empty pGIPZ vector, or shRNA targeting either *Renilla* luciferase or ITCH (2 different ones – 2 and 6) were injected into the tail vein of athymic nude mice. The incidence of metastasis was measured by luciferin injection and bioluminescence imaging. (**C**) Mice were euthanized (day 60) and the lungs from each group of experimental animals were surgically excised, fixed overnight in 10% buffered formalin and metastatic nodules were counted. Data are represented as mean ± standard deviation. The red line signifies the 25th percentile. *P* < 0.001 between the sh-*Renilla* and sh-*ITCH*-2 and *ITCH*-6 groups. (**D, E**) IHC staining for ITCH in lungs obtained from mice described in ‘C’. Brown color indicates positive staining while blue color of the counter stain hematoxylin indicates a negative staining. (**F, G**) IHC staining for YAP in lungs obtained from mice described in ‘C’. Brown color indicates positive staining while blue color of the counter stain hematoxylin indicates a negative staining. Inset in ‘F–G’ shows magnification at 400X.

Based on our *in vitro* findings, we hypothesized that inhibiting *ITCH* expression might impact tumor cell colonization and metastasis. We tested the hypothesis using an *in vivo* xenograft model utilizing tail vein injection. PANC-1 cells were chosen for the xenograft assay because of their high ITCH expression and metastatic potential, compared to the BxPC-3 cells that has low ITCH expression and are non-metastatic. If *ITCH* really has an effect on metastasis, its inhibition would inhibit or attenuate metastatic potential of the parental PANC-1 cells. We first generated PANC-1 cells stably expressing Firefly luciferase (FF+) to make them amenable to *in vivo* live imaging. FF+ PANC-1 cells were transduced with a shRNA targeting *Renilla* luciferase or 6 different shRNAs targeting *ITCH*. Successful knockdown was achieved using *ITCH* shRNA 2 and 6 as verified by immunoblotting (Figure 3A).

*In vivo* luciferase imaging post-tail vein injections showed that whereas PANC-1 cells expressing *Renilla* luciferase shRNA showed profound lung metastasis (Figure 3B), silencing of *ITCH* with either shRNA 2 or 4 significantly inhibited lung colonization (Figure 3B). Furthermore, PANC-1 cells expressing *ITCH* shRNAs showed a significant decrease in numbers of large macrometastases (*P* < 0.001) (Figure 3C).

To confirm that ITCH enhances pancreatic cancer metastasis by inhibiting the Hippo pathway, lung tissues from mice injected with PANC-1 cells harboring shRNA against *Renilla* luciferase and *ITCH* (shRNA-2) were compared for ITCH and YAP expression. As expected, ITCH expression was significantly higher in lungs obtained from animals injected with PANC-1 cells expressing *Renilla* luciferase shRNA as compared to those injected with PANC-1 cells expressing *ITCH* shRNA (Figure 3D, 3E). High expression of ITCH normally signifies low LATS-1 activity and hence higher phosphorylation and nuclear translocation of YAP [4, 7]. Animals injected with *Renilla* shRNA harboring PANC-1 cells, showed predominant strong nuclear staining of YAP in metastatic lung tissue (Figure 3F). However, mice injected with *ITCH* shRNA-2 harboring PANC-1 cells showed exclusive YAP cytoplasmic staining (Figure 3G). Collectively, these results indicate that *ITCH* is of paramount importance in regulating *in vivo* pancreatic cancer metastatic progression and does so by inhibiting Hippo signaling and promoting nuclear translocation of YAP.

### ITCH is a target of miR-106b in pancreatic cancer

**Figure 4:**
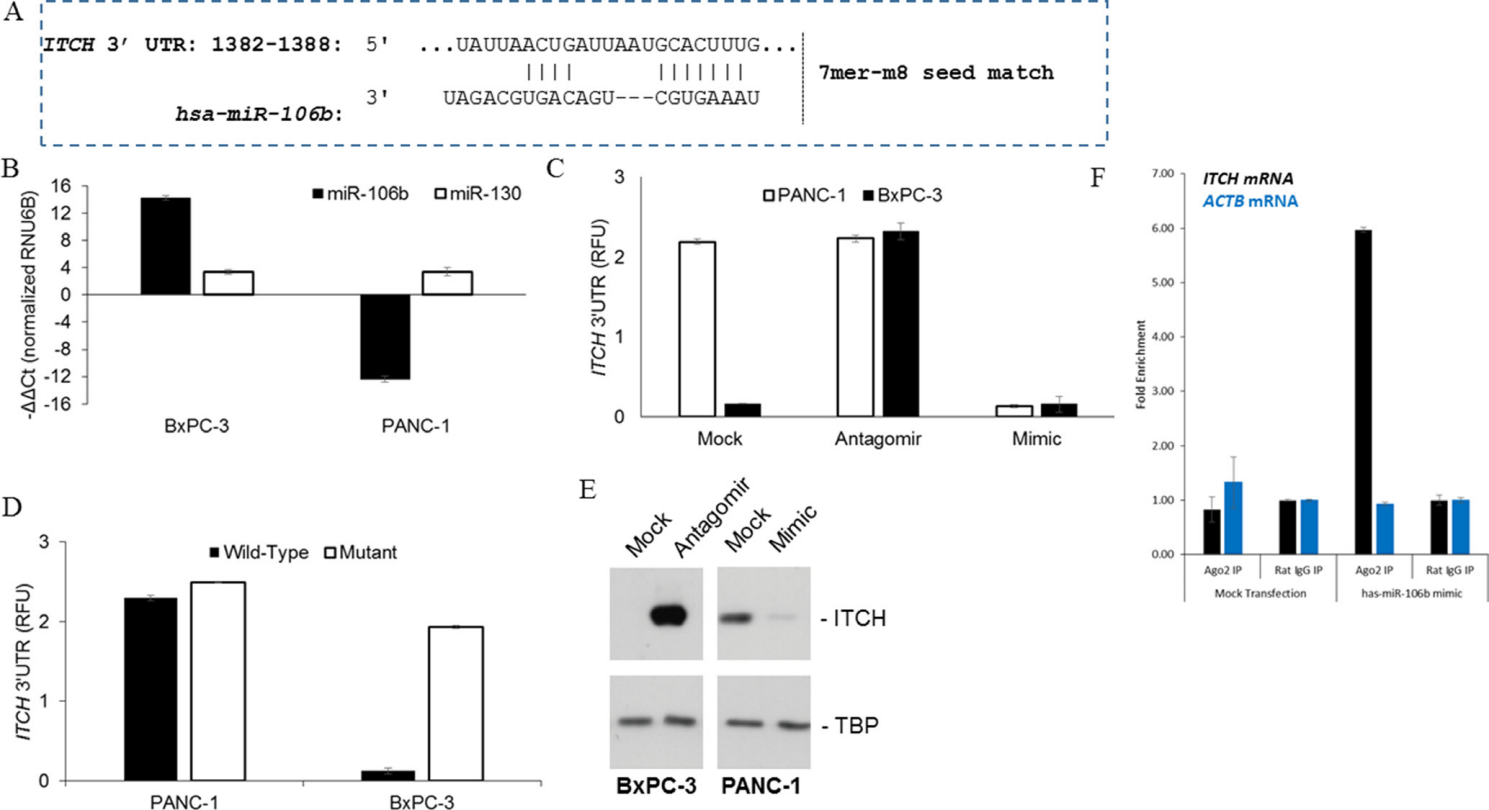
Identification of *ITCH* as a target of miR-106b in pancreatic cancer cells (**A**) Complementary 7mer-m8 seed match between miR-106b and the 3′ UTR of *ITCH* as predicted by TargetScan software. (**B**) Quantitation of miR-106b and miR-130 in BxPC-3 and PANC-1 cell lines. (**C**) Relative luciferase activity of transiently transfected luciferase reporter constructs containing full-length *ITCH* 3′UTR in BxPC-3 and PANC-1 cells, alone or in combination with miR-106b mimic and antagomir. (**D**) Relative luciferase activity of transiently transfected luciferase reporter constructs containing either full-length or mutated (miR-106b binding site deleted) *ITCH* 3′UTR in BxPC-3 and PANC-1 cells. (**E**) Immunoblot analysis of ITCH BxPC-3 and PANC-1 cell line transfected with miR-106b antagomir and mimic, respectively. TBP served as a loading control in each case. (**F**) RNA crosslinking-immunoprecipitation/qRT-PCR of *ITCH* and *ACTB* mRNAs from BxPc-3 cells either mock transfected or transfected with biotinylated *has-miR-106b* mimic using Ago2 antibody or rat IgG and then by streptavidin antibody. *ACTB* is a non-*has-miR-160b* target negative control.

We next wanted to determine what dictates ITCH overexpression in pancreatic cancer tissues. It has been previously indicated [19] that *ITCH* mRNA may be targeted by *hsa-miR-106b* (Figure 4A) in chronic lymphocytic leukemia patients. We hypothesized that if this is the case then miR-106b and ITCH expression should be inversely correlated. Steady state expression of *hsa-miR-106b*, a non-related microRNA (miRNA) *hsa-miR-130*, and endogenous control (*RNU6B*) were determined in BxPC-3 and PANC-1 cells. Data was normalized and expressed relative to *RNU6B*. Whereas there was no detectable difference in *hsa-miR-130* expression between the two cell lines, *hsa-miR-106b* was expressed almost 30 folds higher in BxPC-3 compared to the PANC-1 cell line (*P* < 0.001) (Figure 4B).

We next determined if *ITCH* is a bona fide target of *hsa-miR-106b* in pancreatic cancer cell lines. To test this putative interaction, luciferase reporter constructs containing the wild-type *ITCH* 3′UTR were transfected in BxPC-3 (high *hsa-miR-106b* expression) and PANC-1 (low *hsa-miR-106b* expression) alone or with *hsa-miR-106b* mimic or antagomir (Figure 4C). *ITCH* 3′UTR containing reporter were inhibited 2.3 ± 0.05 folds (*P* = 0.004) in BxPC-3 cells compared to PANC-1 cell line. Co-transfection of *hsa-miR-106b* antagomir rescued the inhibition in BxPC-3 cells, whereas co-transfection of *hsa-miR-106b* mimic caused inhibition of the reporter 2.15 ± 0.25 folds (*P* = 0.001) in the PANC-1 cells (Figure 4C). To confirm that the effects observed was due to *hsa-miR-106b* targeting the *ITCH* 3′UTR, we generated and tested a *hsa-miR-106b* binding mutant of the *ITCH* 3′UTR reporter. The *hsa-miR-106b* binding mutant *ITCH* reporter did not show any difference in relative luciferase activity between PANC-1 and BxPC-3 cells (Figure 4D), confirming that *ITCH* mRNA was being targeted by the *hsa-miR-106b* in these cells. This was further corroborated by immunoblot analysis of BxPC-3 and PANC-1 cell lysates transfected with miR-106b antagomir and mimic, respectively (Figure 4E), which showed that inhibition of *hsa-miR-106b* resulted in ITCH expression in BxPC-3 cells and overexpression of *hsa-miR-106b* in PANC-1 cells caused suppression of ITCH expression in PANC-1 cells. Finally, to determine direct *in vivo* interaction between *hsa-miR-106b* and *ITCH* 3′UTR, we utilized UV crosslinking/immunoprecipitation/qRT-PCR. Post-nuclear lysate from BxPC-3 cells transfected with biotinylated or non-biotinylated *has-miR-106b* mimic were immunoprecipitated first using an anti-Argonaute 2 (Ago2) antibody or rat IgG and then with anti-streptavidin antibody. Ago2 immunoprecipitation, but not rat IgG precipitation, from biotinylated *has-miR-106b* mimic transfected BxPc-3 cells resulted in significant enrichment of *ITCH* mRNA, but not a non-targeted *ACTB* (encoding β-actin) mRNA (Figure 4F). This proved that *has-miR-106b* interacted with the *ITCH* 3′UTR *in vivo*.

### Has-miR-106b expression is inversely correlated with ITCH and metastatic progression in pancreatic cancer patients

**Figure 5:**
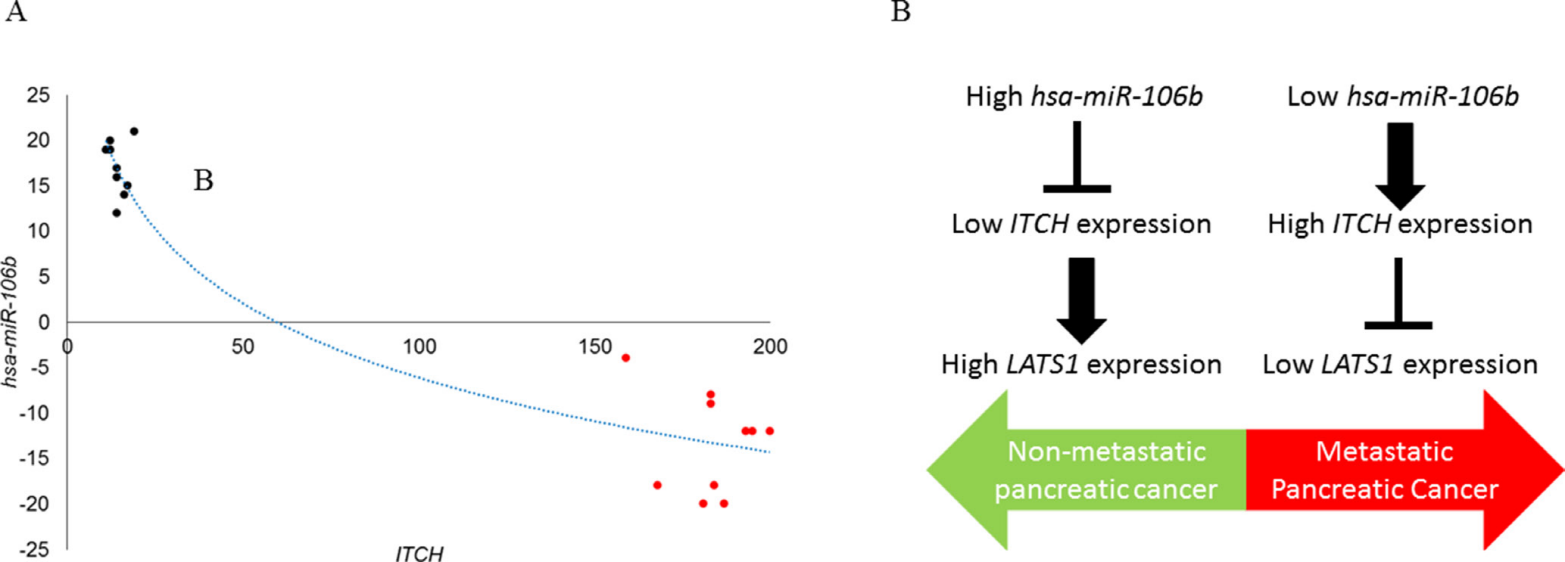
*ITCH* mRNA and miR-106b are inversely correlated in patients with pancreatic cancer (**A**) Pearson correlation demonstrating the inverse relation between miR-106b and ITCH in paired samples (*P* < .005, Pearson correlation *r* = −0.813). (**B**) Schematic representation summarizing the findings from the current study, which shows an inverse correlation expression of miR-106b and *ITCH* in non-metastatic and metastatic human pancreatic cancer.

Given that our experiments indicated a role of *ITCH* in *in vivo* tumorigenesis and metastatic progression and *hsa-miR-106b* mediated regulation of *ITCH*, we hypothesized that suppression of *hsa-miR-106b* expression might be an underlying feature of human pancreatic cancer. To explore the expression of miR-106b and *ITCH* in human pancreatic cancer, we determined miR-106b expression in 20 pancreatic cancer patients, 10 with high *ITCH* and 10 with low *ITCH* expression. Our results indicated an inverse correlation between down-regulation in the levels of *hsa-miR-106b* and the observed increase in the levels of *ITCH* (Figure 5A) (*P* < .005, Pearson correlation *r* = −0.813).

## DISCUSSION

Pancreatic cancer is a heterogeneous disease that widely differ in their pathological characteristics and clinical behavior. Despite treatment advances, almost 60–80% of patients with pancreatic cancer are metastatic at diagnosis [21]. The risk of recurrence is affected by stage at initial presentation and the underlying biology of the tumor. Thus it is imperative to have prognostic and diagnostic markers of human pancreatic cancer that would aid initial detection and also in clinical decision making about treatment modality.

MicroRNAs (miRNAs) are a new class of evolutionarily conserved small RNAs that regulate gene expression at a posttranscriptional level by blocking translation or degrading target messenger RNAs (mRNAs). Growing evidence indicates that microRNAs (miRNAs), a type of endogenous, small noncoding RNAs, participate in diverse cellular processes. Through specifically binding and cleaving mRNAs or inhibiting their translation [22], miRNAs function as either oncogenes or tumor suppressors [23]. Previous studies have indicated that miRNAs are key players in gene regulatory processes and can influence both normal and transformed cellular functionality [21–24]. In fact, miRNAs are reportedly associated with metastatic cancer progression [21, 24]. However, little information regarding the underlying mechanism of miRNAs in pancreatic cancer pathogenesis is available [21]. In that perspective our current finding of miR-160b functioning as a metastatic suppressor by inhibiting ITCH protein expression and regulating Hippo signaling is of paramount significance in advancing the knowledge base (Figure 5B).

Our findings also give certain clues with respect to miRNAs-targeted cancer treatment. Previous studies revealed that some specific miRNAs were often overexpressed in tumors, while most miRNAs were down regulated [25, 26]. Global miRNA suppression was found to boost carcinogenesis in both *in vitro* and *in vivo* models [27], highlighting the pro tumorigenic effects following miRNA loss-of-function. Liang *et al.* observed a sensitizing role of miRNA-302 replacement therapy in breast cancer cells to ionizing radiation [28]. Another recent study reported that viral delivery of *let-7* miRNA could inhibit tumor growth in a mouse lung adenocarcinoma model [29]. Likewise, our results shows the inhibitory effect of *hsa-miR-106b* in pancreatic cancer cells. Taken together, these studies suggest that overexpression of even a single miRNA in cancer cells might confer substantial therapeutic benefit.

In summary, we have shown that *ITCH* over expression and *has-miR-10b* down regulation are potential novel biomarkers for predicting poor prognosis for human pancreatic cancer. The experiments using BxPC-3 and PANC-1 cell lines further demonstrated that ITCH promotes metastatic progression and its expression is directly regulated by *hsa-miR-106b* expression. It needs to be determined if *ITCH* plays a role in pancreatic cancer tumorigenesis also or whether its function is limited to metastatic progression. It would be interesting to identify what potentially causes downregulation of *hsa-miR-106b* in pancreatic cancer cells and whether the down regulation happens during initial tumorigenesis or only during metastatic progression.

## MATERIALS AND METHODS

### Clinical samples, tissue processing, and ethical considerations

Fresh-frozen and paraffin-embedded pancreatic cancer tissues and corresponding adjacent non-tumorous pancreatic tissue samples were obtained from 30 Chinese patients at Chengdu Military General Hospital between 2008 and 2010. All cases were included post review by pathologist and histological confirmation as pancreatic cancer and only where complete clinical pathology and follow-up data was available. None of the 30 included patients underwent pre-operative local or systemic treatment. The study protocol was approved by the Institutional Review Board of the Chengdu Military General Hospital, China. Freshly harvested samples were immersed in RNAlater (Life Technologies, Gaithersburg, MD, USA) before snap freezing within 30 minutes post-surgery. All tissue samples were stored in liquid nitrogen until further use.

### Cell culture

BxPC-3 and PANC-1 cell lines were obtained from the Cell Bank of Shanghai Institute of Biochemistry & Cell Biology, Chinese Academy of Sciences (Shanghai, China) (in October 2013). Cell lines were characterized in the parent bank by the STR method (in July 2013) and fresh vials were used every six months post-resuscitation. All cell lines were routinely tested for mycoplasma contamination and verified to be Mycoplasma free (last control November 2014). BxPC-3 cells were grown in RPMI 1640 with 10% fetal bovine serum (FBS) (Sigma Aldrich, St. Louis., MO, USA). PANC-1 cells were grown as a monolayer cell culture in DMEM containing 4.5 mg/mL D-glucose and L-glutamine (Life Technologies, Gaithersburg, MD, USA) supplemented with 10% FBS. Cells were kept at 37°C under a humidified atmosphere of 5% carbon dioxide.

### RNA and miRNA extraction and quantitative real time polymerase chain reaction (PCR)

Total RNA was isolated from cultured cells and tumor tissues using Trizol reagent (Life Technologies, Gaithersburg, MD, USA). First strand cDNA was synthesized using the RevertAid™ First Strand cDNA synthesis Kit (Life Technologies, Gaithersburg, MD, USA), which was then used for real-time polymerase chain reaction (PCR) using TaqMan Gene Expression probes (Life Technologies, Gaithersburg, MD, USA). *TBP* (TaqMan Assay ID: Hs00427620_m1) was used as an internal control for *ITCH* (TaqMan Assay ID: Hs00395201_m1) and *LATS1* (TaqMan Assay ID: Hs01125523_m1) transcript levels. Data was normalized to *TBP* expression and analyzed by the −ΔΔCt method.

According to the manufacturer's instructions, miRNA from tissues and cells was extracted using the mirVana miRNA isolation kit (Life Technologies, Gaithersburg, MD, USA), and the expression levels of miRNA-16ba and miR-130 were detected by TaqMan miRNA assays (Life Technologies, Gaithersburg, MD, USA), using U6 small nuclear RNA as an internal control.

### Preparation of whole cell lysates and immunoblot analysis

Cells were lysed in buffer containing 25 mM Tris-HCl pH 7.4, 150 mM NaCl, 1 mM EDTA, 1% NP-40 and 5% glycerol containing complete, Mini protease inhibitor cocktail (Roche Diagnostics, Indianapolis, USA). Ten micrograms of whole cell lysate was resolved on a NuPAGE 4–20% gel (Life Technologies, Gaithersburg, MD, USA), transferred to an Immobilon PVDF membrane (Millipore, Billerica, USA), and probed with LATS-1 and ITCH antibodies (Abcam, Cambridge, USA). The blot was subsequently stripped, and re-probed for TBP (Cell Signaling, Danvers, USA) to confirm equal loading. The blots were imaged using ECL Plus western blotting substrate and autoradiography film.

### Subcloning of luciferase reporter constructs

The *ITCH* 3′UTR reporter was constructed by amplifying the endogenous *ITCH* 3′UTR from Hela cell genomic DNA using primers described earlier [19]. *Xho*I and *Apa*I sites were added to the 5′ and 3′ ends of the fragment during the preceding PCR reaction and cloned into the *Xho*I and *Apa*I site on the *Rr-luc-6XCXCR4* (Addgene plasmid 11308) Renilla luciferase vector. To make the *ITCH* 3′UTR mutant construct, site-directed mutagenesis was used to delete 1382–1388 region, corresponding to the *has-miR-106b* binding site. A firefly luciferase vector was used as transfection and normalization control in all luciferase assays. Constructs were sequence verified to UCSC human genome reference version hg19.

### Transfection and luciferase assays

PANC-1 and BxPC-3 cells (4 × 10^4^) were transiently transfected using Lipofectamine LTX (Life Technologies, Gaithersburg, MD, USA) as per the manufacturer's instructions. Where indicated, 300 nM mimics and antagomirs (inhibitors) (Life Technologies, Gaithersburg, MD, USA) were transfected along with the *ITCH* 3′UTR constructs. Forty-eight hours after transfection, the renilla and firefly luciferase activities were measured consecutively using Dual-luciferase reporter assay system (Promega, Madison, Wisconsin, USA) as per manufacturer's protocol. Each reporter plasmid was transfected at least twice (on different days) in triplicate. Post-normalization the data was represented as relative fluorescence units (RFU) ± standard deviation (SD).

### Recombinant lentivirion production, and stable cell lines generation

293T (4 × 10^4^ cells) were transiently transfected using Lipofectamine LTX (Life Technologies, Gaithersburg, MD, USA) as per the manufacturer's instructions. PANC-1 cells were transduced with the lentiviral soup containing Firefly Luciferase (FF+) (AMS Biotechnology, Lake Forest, CA, USA) and stable transductants were selected using Blastocidin (5 μg/ml). The *pGFP-V-RS* (containing 6 different shRNAs (Origene, Rockville, MD, USA) lentiviral particles were generated by transfection of 293Ts using Mirus Transit-293T. PANC-1 FF+ cells transduced with *pGFP-V-RS-shRNA-ITCH* or *Renilla* were selected with Puromycin (2 μg/mL) to generate stable pools. In all cases, gene silencing was verified both by visualization of GFP expression and by immunoblotting.

### Xenograft assays

All animal procedures were approved by the Institutional Animal Care and Use Committees of the Chengdu Military General Hospital. Six weeks old spontaneous mutant T cell deficient female homozygous nude mice (CrTac:NCr-Foxn1^nu^) (Taconic Farms Inc., Germantown, NY, USA) were used. To assess the metastatic potential of cells, 1 × 10^6^ FF+ PANC1-*Renilla* luciferase shRNA or FF+ PANC1-*ITCH* shRNA-2 and shRNA-6 cells were injected into nude mice (*n* = 5 per group) via the tail vein. Mice were assessed weekly for metastasis using *in vivo* bioluminescence imaging using an IVIS Imaging System (IVIS imaging system 200, Xenogen Corporation, PerkinElmer, Waltham, USA) fitted with an ultrasensitive CCD camera. Camera resolution was set at medium sensitivity and D-luciferin (Xenogen) was injected intraperitoneally at 3 mg/mouse 10 minutes before imaging. Mice were monitored for lung metastasis and euthanized after 8 weeks, at which time the lungs were surgically removed and fixed using 10% neutral buffered formalin and the number of lung tumor nodules was counted using a dissection microscope.

### Immunohistochemistry

Paraffin-embedded lung tissue sections obtained from above were deparaffinized with xylene and rehydrated with step-down changes of ethanol. Antigen retrieval was performed in pre-heated 10 mM sodium citrate buffer pH 6.0 using pressurized for 15 minutes. Endogenous peroxidase was blocked with 3% H_2_O_2_ for 10 minutes. The sections were then incubated with blocking solution for 30 minutes to reduce non-specific binding followed by incubation with the primary antibody (monoclonal anti-ITCH antibody (Sigma Aldrich, St. Louis., MO, USA) [dilution of 1:100], and anti-YAP (Epitomics, Burlingame, CA, USA) [dilution of 1:200]), at 4°C in a humidified chamber for overnight incubation. Post-washing, slides were subsequently incubated with horseradish peroxidase-conjugated antibody for 30 minutes. Detected was done using a freshly prepared 3, 3 diamminobenzidine tetrahydrochloride using DAKO Liquid DAB Substrate-Chromogen (Dako Corporation, Carpinteria, California, USA) solution for several minutes at room temperature.

### RNA immunoprecipitation

BxPc-3 cells were mock transfected or transfected with biotinylated *has-miR-106b* mimic. Forty-eight hours post-transfection, cells were placed on ice, irradiated once with 150 mJ/cm^2^ at 254 nm using a UV Crosslinker (Spectroline, Westbury, NY) and lysed for 15 minutes on ice in lysis buffer. Five mg of of post-nuclear extracts were subjected to immunoprecipitation using 10 μg of Ago2 antibody (clone 11A9, EMD Millipore, Billerica, MA, USA) or 10 μg of rat IgG using Pierce Crosslink IP Kit (Thermo Scientific, Pittsburgh, PA). The rest of the immunoprecipitated complex was digested with 30 μg of proteinase K to release the ribonucleoprotein complex and a second round of immunoprecipitation was performed using anti-streptavidin antibody (EMD Millipore). RNA extraction and qRT-PCR was done as described above. Relative enrichment was calculated from the qRT-PCR data.

### Statistical analyses

The difference between continuous variables was analyzed using the Student's *t*-test or analysis of variance. Two-sided *P*-values < 0.05 were considered statistically significant. Statistical analyses were performed using SPSS version 20.0 (IBM Corporation, NY). Estimates of overall survival (OS) were calculated using Kaplan-Meier curves. Differences OS between those with high and low ITCH expression were determined using the log-rank test for equality of survival functions (Prism v 5.0, GraphPad, La Jolla, California, USA). Finally, Cox regression analyses (Stata 12.1, StataCorp, College Station, Texas, USA) were used in order to calculate hazard ratios (HRs) and 95% confidence intervals (CIs) to determine the association between ITCH expression and progression after adjusting for age and gender.
